# A missense mutation in solute carrier family 12, member 1 (*SLC12A1*) causes hydrallantois in Japanese Black cattle

**DOI:** 10.1186/s12864-016-3035-1

**Published:** 2016-09-09

**Authors:** Shinji Sasaki, Kiyotoshi Hasegawa, Tomoko Higashi, Yutaka Suzuki, Sumio Sugano, Yasuaki Yasuda, Yoshikazu Sugimoto

**Affiliations:** 1 Shirakawa Institute of Animal Genetics, Japan Livestock Technology Association, Odakura, Nishigo, Fukushima 961-8061 Japan; 2 Shimane Prefecture Livestock Technology Center, Koshi, Izumo, Shimane 693-0031 Japan; 3 Shimane Prefecture Livestock Division Livestock Hygiene Research Office, Jinzaioki, Izumo, Shimane 699-0822 Japan; 4 Department of Medical Genome Sciences, and Department of Computational Biology, Graduate School of Frontier Sciences, The University of Tokyo, Chiba, 277-8562 Japan

**Keywords:** Hydrallantois (hydrops allantois), Solute carrier family 12, member 1 (*SLC12A1*), Na-K-2Cl cotransporter (NKCC2), Bartter syndrome, Counter current mechanism, Autosomal recessive disorder, Autozygosity mapping, Exome sequencing, Japanese Black cattle

## Abstract

**Background:**

Hydrallantois is the excessive accumulation of fluid within the allantoic cavity in pregnant animals and is associated with fetal mortality. Although the incidence of hydrallantois is very low in artificial insemination breeding programs in cattle, recently 38 cows with the phenotypic appearance of hydrallantois were reported in a local subpopulation of Japanese Black cattle. Of these, 33 were traced back to the same sire; however, both their parents were reported healthy, suggesting that hydrallantois is a recessive inherited disorder. To identify autozygous chromosome segments shared by individuals with hydrallantois and the causative mutation in Japanese Black cattle, we performed autozygosity mapping using single-nucleotide polymorphism (SNP) array and exome sequencing.

**Results:**

Shared haplotypes of the affected fetuses spanned 3.52 Mb on bovine chromosome 10. Exome sequencing identified a SNP (g.62382825G > A, p.Pro372Leu) in exon 10 of solute carrier family 12, member 1 (*SLC12A1*), the genotype of which was compatible with recessive inheritance. SLC12A1 serves as a reabsorption molecule of Na^+^-K^+^-2Cl^−^ in the apical membrane of the thick ascending limb of the loop of Henle in the kidney. We observed that the concentration of Na^+^-Cl^−^ increased in allantoic fluid of homozygous *SLC12A1* (g.62382825G > A) in a hydrallantois individual. In addition, SLC12A1-positive signals were localized at the apical membrane in the kidneys of unaffected fetuses, whereas they were absent from the apical membrane in the kidneys of affected fetuses. These results suggested that p.Pro372Leu affects the membrane localization of SLC12A1, and in turn, may impair its transporter activity. Surveillance of the risk-allele frequency revealed that the carriers were restricted to the local subpopulation of Japanese Black cattle. Moreover, we identified a founder individual that carried the mutation (g.62382825G > A).

**Conclusions:**

In this study, we mapped the shared haplotypes of affected fetuses using autozygosity mapping and identified a de novo mutation in the *SLC12A1* gene that was associated with hydrallantois in Japanese Black cattle. In kidneys of hydrallantois-affected fetuses, the mutation in *SLC12A1* impaired the apical membrane localization of SLC12A1 and reabsorption of Na^+^-K^+^-2Cl^−^ in the thick ascending limb of the loop of Henle, leading to a defect in the concentration of urine via the countercurrent mechanism. Consequently, the affected fetuses exhibited polyuria that accumulated in the allantoic cavity. Surveillance of the risk-allele frequency indicated that carriers were not widespread throughout the Japanese Black cattle population. Moreover, we identified the founder individual, and thus could effectively manage the disorder in the population.

**Electronic supplementary material:**

The online version of this article (doi:10.1186/s12864-016-3035-1) contains supplementary material, which is available to authorized users.

## Background

Hydrallantois (hydrops allantois) is the excessive accumulation of fluid within the allantoic cavity in pregnant animals. Allantoic fluid volume in affected cows may exceed 100 L [1–3], although only 2–3 L is normal during mid-gestation (5–7 months: 152–213 days) [3]. This leads to rapid abdominal enlargement of the dam within 1 month after the appearance of clinical signs [1]. Poor prognosis for the affected fetuses and mothers often necessitates induced delivery (for review, see [1, 2, 4]).

Hydrallantois is commonly observed in cows carrying twin fetuses, those with severe nutritional deficiency (for review, see [4]), and pregnancies with in vitro fertilized (IVF) [5, 6] and somatic nuclear transferred embryos [7]. Meanwhile, the frequency of hydrallantois in cows fertilized by natural or artificial insemination (AI) is extremely low: it is approximately 0.01 % for non-IVF pregnancies [8] and for beef cattle in Japan [9]. Between 2009 and 2014, however, hydrallantois was observed in 38 Japanese Black cattle (0.084 % of 45,801 cows) in AI breeding programs in a local subpopulation in Japan [10]. The fetuses were aborted and 45 % of the cases were associated with maternal death, compromising the welfare of animal, resulting in severe economic losses for individual farmers. Pedigree analysis indicated that the parents of 33 affected individuals were traced back to the same elite sire, however, these parents were not affected by hydrallantois during the fetal period and were reported healthy, suggesting that hydrallantois is a recessive inherited fetal disorder. This highlights one of the problems of AI, where a limited number of sires is used, exposing recessive genetic defects that rapidly spread throughout a subpopulation. Thus, identifying the causative mutation and developing a gene diagnostic test is essential to identify and exclude carrier individuals from breeding programs. Although the incidence of hydrallantois in other cattle breeds has been reported [11–13], the inheritance patterns and genetic factors related to this condition have not been evaluated and identified in cattle.

In the present study, we performed autozygosity mapping using single-nucleotide polymorphism (SNP) array [14, 15] and exome sequencing to identify autozygous chromosome segments shared by animals with hydrallantois and the causative mutation in Japanese Black cattle. We identified a missense mutation in solute carrier family 12, member 1 (*SLC12A1*), which serves as a key regulator, controlling the urine concentration via a countercurrent mechanism in the kidneys.

## Results and Discussion

### A 3.52 Mb autozygous segment on chromosome 10 was shared by hydrallantois-affected fetuses

**Fig. 1:**
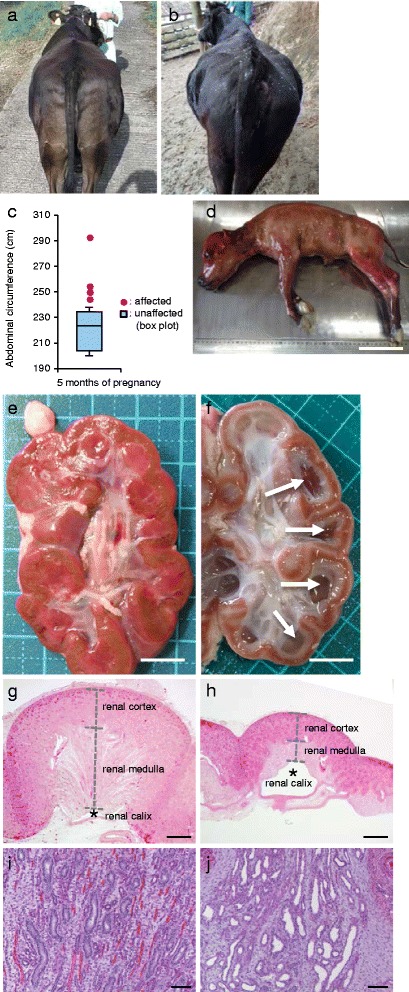
Symptoms of hydrallantois in Japanese Black cattle. **a**, **b** Caudal views of an unaffected cow (**a**) and hydrallantois-affected cow (**b**) at 5 months (152 days) of pregnancy. Image shows abdominal enlargement in the affected cow (**b**) relative to the unaffected cow (**a**). **c** Abdominal circumference of the affected cows was significantly longer than that of unaffected cows at 5 months (152 days) of pregnancy (*t*-test, *P* = 0.0144). **d** Hydrallantois-affected fetus at approximately 160 days of gestation, estimated from crown-rump length (CRL). **e**, **f** Images of the sagittal section of the kidney in unaffected (**e**) and affected fetus (**f**) at approximately 160 days of gestation. Hydronephrosis was associated with dilation of the calices (*white*-*arrows*, **f**) in an affected fetus. **g**, **h**, **i**, **j** Micrographs of hematoxylin eosin-stained kidney sections from an unaffected fetus (**g**, **i**) and an affected fetus (**h**, **j**) at approximately 160 days of gestation. Atrophy of the renal cortex and medulla of kidneys in an affected fetus (**h**). Dilation of collecting ducts in the medulla of kidneys in an affected fetus (**j**). Scale bars: **d** = 10 cm; **e**, **f** = 1 cm; **g**, **h** = 1 mm; **i**, **j** = 100 μm

Between October 2014 and April 2015, we obtained biological materials and clinical information with pedigree records of nine Japanese Black cattle that were diagnosed with hydrallantois (for diagnostic criteria, see [1]). The affected cows exhibited rapid abdominal enlargement within 1 month of diagnosis between 5.2 months (158 days) and 9.2 months (280 days) of pregnancy (Fig. 1a, b). The abdominal circumference of the affected cows was significantly longer than that of the unaffected cows at 5 months (152 days) of pregnancy (*t*-test, *P* = 0.0144, Fig. 1c). In all the cases, the affected fetus was a singleton. Aborted fetuses were grossly normal, without malformation and did not show symptoms of dropsy (Fig. 1d). At necropsy, we observed that hydronephrosis, which was associated with dilation of the renal calices (Fig. 1e–h) and atrophy of the renal cortex and medulla of the kidney (Fig. 1g, h). Histopathological examination revealed that the convoluted tubules and collecting ducts were dilated (Fig. 1i, j), suggesting that the outflow of urine was obstructed.

**Fig. 2:**
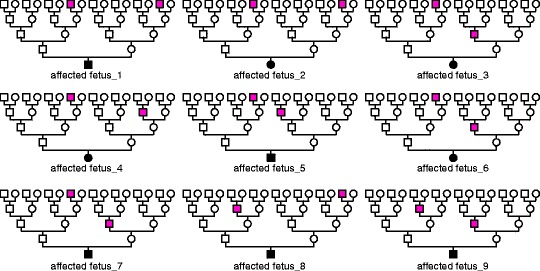
Pedigree analysis of hydrallantois-affected fetuses in Japanese Black cattle. Family tree showing nine hydrallantois-affected fetuses traced back two to four generations on both paternal and maternal sides to an ancestor; sire_X (magenta-filled square). Males are represented by squares, and females by circles. Affected individuals are indicated with black-filled symbols

Examination of the pedigree of the nine cattle suggested a recessive mode of inheritance as all the affected individuals could be traced back for two to four generations on both paternal and maternal sides to a common ancestor, sire_X (magenta-filled squares in Fig. 2). We performed PCR using a primer specific for the Y chromosome to determine the sex of the affected fetuses (Additional file 1: Table S1). Results showed that the sex ratio of males to females was 5:4 (﻿Fig. 2, Additional file 2); there was, therefore, no sex bias in the affected fetuses, suggesting that hydrallantois is an autosomal recessive inherited disorder.

**Fig. 3:**
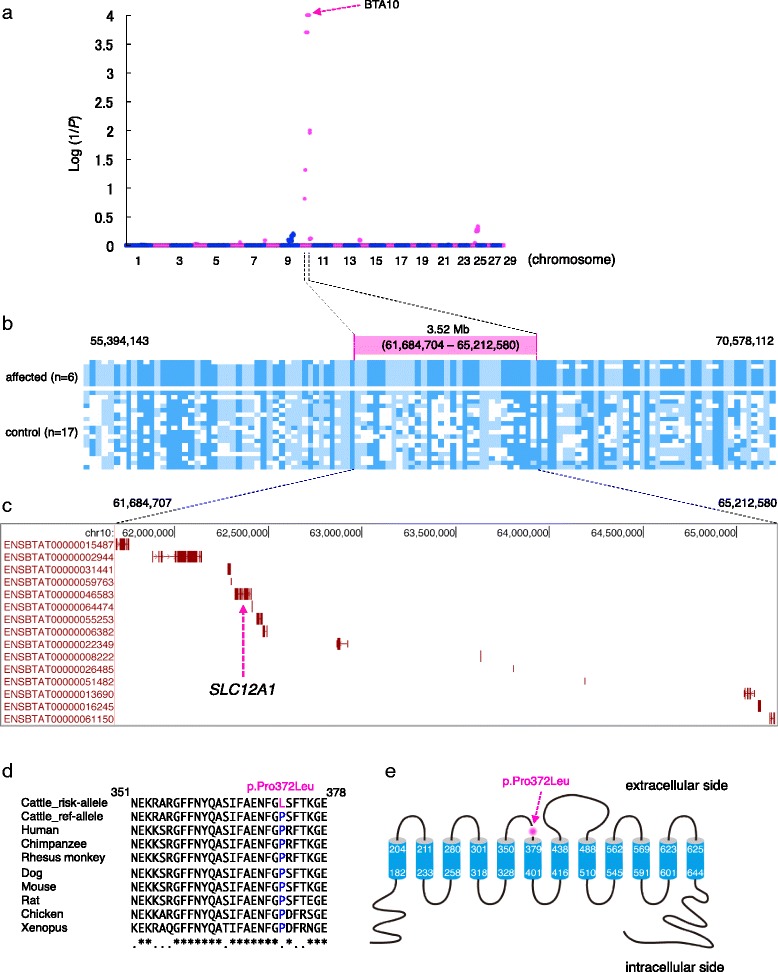
Hydrallantois locus in a 3.52 Mb segment on bovine chromosome 10 and missense mutation (p.Pro372Leu) in *SLC12A1* **a** Autozygosity mapping for the hydrallantois locus on bovine chromosome 10 (BTA10) using ASSIST program. Blue and magenta dots measure the genome-wide probability that the six hydrallantois-affected fetuses share the segment of autozygosity (blue, odd numbers of chromosomes; magenta, even numbers of chromosomes). Evidence for linkage (y-axis) is measured as log (1/*P*), with *P* being determined by 10,000 phenotype permutation tests. **b** Genotype of six affected and 17 unaffected calves from 55,394,143 to 70,578,112 bp on BTA10. Homozygous genotypes are shown in blue (AA), and light blue (BB), and heterozygous genotypes in white. A homozygous segment encompassing the hydrallantois locus in the six affected individuals is shown with a magenta bar. **c** Gene content of the 3.52 Mb autozygous segment. Ensembl transcript IDs were labeled. **d** Cattle SLC12A1 and interspecies alignments of the region flanking p.Pro372Leu (magenta), showing conservation of the p.Pro372 residue (blue). (E) SLC12A1 is a 12 membrane-spanning transmembrane protein. The p.Pro372Leu resides (*magenta arrow*) within the third portion of the predicted extracellular side

We obtained good-quality, high-molecular weight DNA from six affected fetuses; however, the DNA from three cases had degraded into small sizes (<100 bp), probably because of the time that elapsed between fetal death and sampling. Autozygosity mapping using the ASSIST program to search for shared homozygous and identical-by-state chromosome segments among the affected individuals [16], identified a single, genome-wide significant peak (*P* < 0.0001) on the bovine chromosome 10 (BTA10) (Fig. 3a). The shared haplotype spanned 3.52 Mb (61,684,704–65,212,580 bp) (Fig. 3b, Additional file 1: Table S2). All parents of the nine affected fetuses and sire_X had the heterozygous risk-haplotype, which is consistent with the autosomal recessive model.

There was no evidence for the presence of copy number variation (CNV) in the six affected fetuses, or risk-haplotype carrier individuals within 61,684,704–65,212,580 bp. In addition, although we detected five CNVs in the risk-haplotype region from the CNVs dataset of 1481 Japanese Black cattle [17], these CNV-detected individuals did not have the risk-haplotype (Additional file 1: Table S3). Thus, these results suggested that the causative mutation was not a large structural variant.

### Exome sequencing to identify a candidate causative mutation in solute carrier family 12, member 1 (*SLC12A1*) gene

The shared haplotype region contained 15 annotated transcripts (Fig. 3c, Additional file 1: Table S4). To identify the underlying mutation responsible for hydrallantois, we sequenced the exomes of two affected fetuses with the homozygous risk-haplotype and two unaffected calves without the risk-haplotype. Alignment of filtered sequence reads resulted in 52- and 48-fold read depth coverage of the targets in the affected fetuses, and 42- and 50-fold read depth coverage of the targets in the unaffected calves, respectively. Alignment of the sequence reads from all the four individuals against the risk-haplotype region identified 439 sequence variations (408 SNPs and 31 indels). Among them, 32 SNPs and one indel were compatible with the recessive inheritance that was homozygous for the non-reference allele in the two affected fetuses and homozygous for the reference allele in the two unaffected calves. Of the 33 variants, two SNPs were missense mutations in the coding regions, which was subsequently confirmed by Sanger sequencing (Table 1. Additional file 1: Table S5). Among these, one SNP (g.62382825G > A, p.Pro372Leu was based on XM_005211900 [Additional file 1: Table S6]) in exon 10 of *SLC12A1* was fully compatible with recessive inheritance that was homozygous for the non-reference allele in the two affected fetuses, heterozygous for the allele in the two carrier calves (heterozygous for the risk-haplotype), and homozygous for the reference allele in the two unaffected calves (Additional file 1: Table S7). Consistent with these data, all the parents (sires and dams) of the nine affected fetuses and sire_X (Fig. 2) were heterozygous for the risk-allele (g.62382825A). To search for other variants in *SLC12A1*, we sequenced all the exons (exons 1–28) of *SLC12A1* in two affected fetuses homozygous for the risk-haplotype, two carrier dams, and two unaffected calves without the risk-haplotype (primer details are provided in Additional file 1: Table S8). Except for the SNP (g.62382825G > A) in exon 10, we did not detect any variants in *SLC12A1*. The p.Pro372Leu amino acid substitution in *SLC12A1* was predicted as probably damaging by Polyphen-2 [18] and deleterious by SIFT [19] (Table 1), which are programs for protein-sequence-based prediction of deleteriousness of a mutation for protein function. The cattle gene *SLC12A1*, which was based on XM_005211900 (Additional file 1: Table S6), encodes a 12 membrane-spanning protein containing 1099 amino acids. p.Pro372 is highly conserved among the species (Fig. 3d) and is located on the extracellular side (Fig. 3e), which was predicted by SMART [20].

**Table 1:** Two missense variants in the shared haplotype region of hydrallantois as revealed by exome sequencing

				Polyphen-2^b^		SIFT^c^	
Chr	SNP^a^	Gene symbol	Substitution	Prediction	Score	Prediction	Score
10	g.62111230G > A	*FBN1*	p.V2290I	benign	0	tolerated	0.36
10	g.62382825G > A	*SLC12A1*	p.P372L	probably damaging	0.993	deleterious	0

Exome sequencing of two affected fetuses with homozygous risk-haplotype and two unaffected calves without risk-haplotype

^a^Positions are based on the UMD3.1 assembly of the bovine genome

^b^Polyphen-2 score represents the probability that a substitution is deleterious, with values nearer 1 indicating more confident predictions

^c^SIFT score is the normalized probability that a substitution is tolerated, with values nearer 0 being more likely to be deleterious

SLC12A1 serves as a reabsorption molecule of Na^+^-K^+^-2Cl^−^ in the thick ascending limb of the loop of Henle in the kidney (for review, see [21]), which induces reabsorption of water via the countercurrent mechanism and concentrates urine [22]. The homozygous loss-of-function mutation of *SLC12A1* in humans is responsible for the antenatal form of Bartter syndrome type I [23–26], which is an autosomal recessive disorder characterized by fetal polyuria and hydramnios that closely resemble the symptoms of hydrallantois in Japanese Black cattle. Skydsgaard reported that the concentration of Na^+^-Cl^−^ in the allantoic fluid of Red Danish and Jersey cow with hydrallantois (Na^+^ = 116 ± 13 mEq/L, Cl^−^ = 81 ± 12 mEq/L) at 7–9 months (213–274 days) gestation was higher than that in the unaffected cows (Na^+^ = 52 ± 20 mEq/L, Cl^−^ = 17 ± 11 mEq/L) [13]. Consistent with this, we also observed that the concentration of Na^+^-Cl^−^ increased in the allantoic fluid of a cow homozygous for *SLC12A1* (﻿g.62382825G > A, p.Pro372Leu) with hydrallantois at 5.4 months (164 days) gestation (Na^+^ = 130 mEq/L, Cl^−^ = 96 mEq/L), which may reflect an impairment of Na^+^ reabsorption and the presence of Na^+^ waste in the urine, because the fetal urine accumulated in the allantoic cavity. Thus, we considered a mutation in *SLC12A1* (g.62382825G > A, p.Pro372Leu) as a candidate causative mutation.

### Localization of SLC12A1 at the apical membrane of the thick ascending limb of the loop of Henle and distal convoluted tubules in kidneys of cattle fetuses

**Fig. 4:**
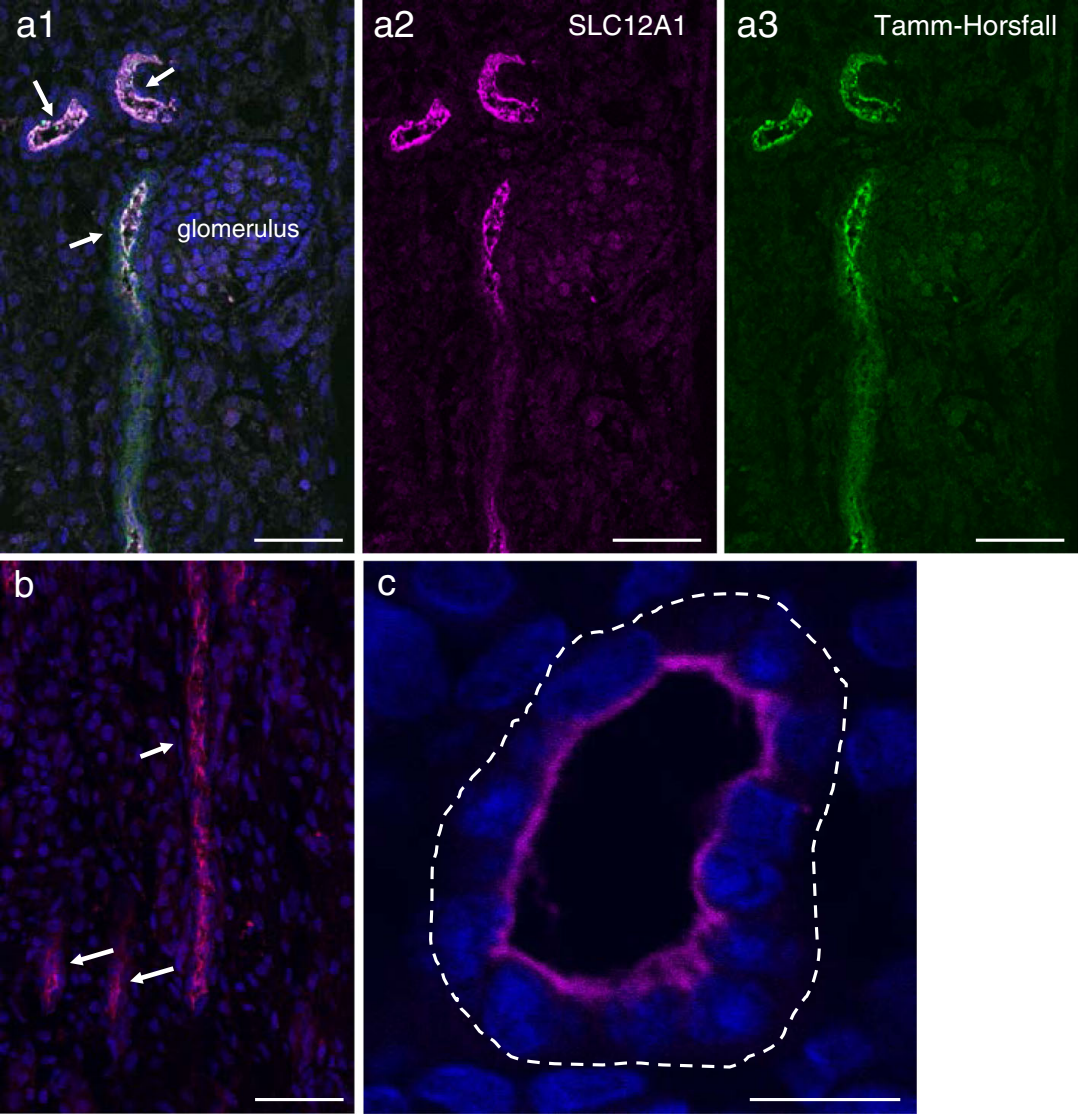
Localization of SLC12A1 protein in fetal kidney of Japanese Black cattle. **a**, **b**, **c** Localization of SLC12A1 in the kidney of fetus of 46-cm crown-rump length (CRL), approximately 160 days of gestation. Sections were immunostained with anti-SLC12A1 antibody (**a1**, **a2**, **b**, **c**; magenta) and anti-Tamm-Horsfall antibody (**a1**, **a3**; green), and counterstained with DAPI (**a1**, **b**, **c**). Images **a** and **b** show the renal cortex and medulla, respectively. **a1** Distal convoluted tubules are indicated by arrows. **b** Thick limbs of the loop of Henle are indicated by arrows. Image **c** shows higher magnification of a distal convoluted tubule (SLC12A1; magenta). Basal membrane side is indicated by dotted-line circle. Scale bars: **a**, **b** = 50 μm; **c** = 10 μm

We applied double immunostaining with antibody to SLC12A1 and Tamm-Horsfall (TH) glycoprotein as a marker for the thick limb of the loop of Henle and distal convoluted tubules [27, 28]. Results showed that SLC12A1-positive segments of tubules were almost all TH-positive, indicating that SLC12A1 is localized in both the thick limb of the loop of Henle and distal convoluted tubules in the kidneys of cattle fetuses of 46 cm crown-rump length (CRL), at approximately 160 days of gestation [29] (Fig. 4a, b). Higher magnification images revealed that the SLC12A1-positive signals were localized at the apical membrane of the distal convoluted tubules (Fig. 4c). These results suggest that SLC12A1 may function in cattle fetuses at mid-gestation, as is the case in humans [23–26] and mice [30].

### Effects of SLC12A1 (p.Pro372Leu) on intracellular localization

**Fig. 5:**
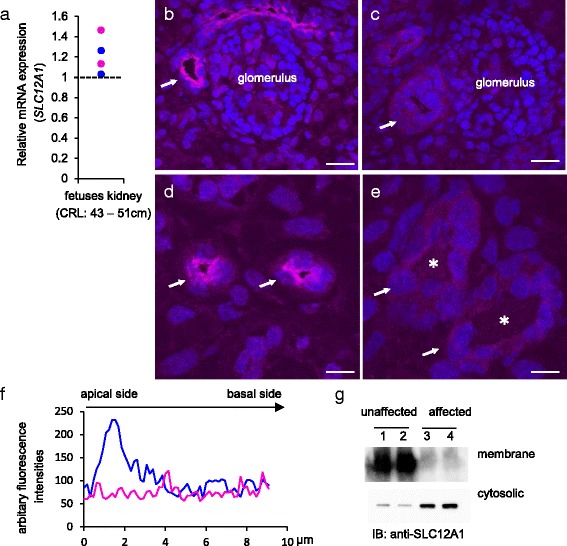
Localization of SLC12A1 in the affected fetal kidneys in Japanese Black cattle. **a** Total RNA was extracted from kidneys of the unaffected (*blue dots*) and affected (*magenta dots*) fetuses at approximately 160 days of gestation, and the unaffected adults for real-time quantitative PCR. Relative gene expression levels in the different samples are shown as mean quantities relative to the value observed in the adult kidney (*dotted line*). **b**, **c**, **d**, **e** Localization of SLC12A1 in the kidneys of unaffected (**b**, **d**) and affected (**c**, **e**) fetuses at approximately 160 days of gestation. Sections were immunostained with anti-SLC12A1 antibody (*magenta*) and counterstained with DAPI (*blue*). Images **b**, **c** and **d**, **e** show the renal cortex and medulla, respectively. **b**, **c** Distal convoluted tubule indicated by arrow. **d**, **e** Thick limbs of the loop of Henle are indicated by arrows. **e** Dilated thick limbs of the loop of Henle in affected fetuses are indicated by asterisks. **f** Line plots of fluorescence intensities from apical to basal side of distal convoluted tubule in the unaffected (*blue line*, **b**) and affected fetuses (*magenta line*, **c**). **g** Membrane and cytosolic fractions were prepared by centrifugation from the unaffected (lanes 1 and 2) and affected (lanes 3 and 4) fetuses at 100,000 × *g* and the immunoblot (IB) was performed using anti-SLC12A1 antibody. Scale bars: **b**, **c** = 20 μm; **d**, **e** = 10 μm

We investigated the functional consequences of p.Pro372Leu in SLC12A1 in the affected fetal kidney. We found that *SLC12A1* mRNA was expressed in the affected fetal kidneys; however, we did not observe obvious quantitative differences between the affected and unaffected fetuses of 43–51 cm CRL, at approximately 160 days of gestation [29] (Fig. 5a).

There was a close relationship between the presence of SLC12A1 at the apical membrane and the transporter activity [31] (for review, see [32]). The localization of SLC12A1 at the apical membrane is determined by exocytic delivery, recycling, and endocytosis (for review, see [32, 33]), which are tightly regulated by domain and posttranslational modifications [31]. To determine whether p.Pro372Leu influences intracellular localization of SLC12A1, we examined the subcellular localization of SLC12A1 in the fetal kidneys using an anti-SLC12A1 antibody. The results showed that the SLC12A1-positive signals were not detected at the apical membranes in either the dilated distal convoluted tubules (Fig. 5c) or in the thick limb of the loop of Henle (Fig. 5e) in kidneys of the affected fetuses. We also found that the signals were preferentially localized at the apical side of the unaffected cells, whereas they were weakly and diffusely distributed throughout the affected cells, as shown by semi-quantified fluorescence (Fig. 5f).

To obtain further information on the localization, we assessed SLC12A1 distribution in the membranes (100,000 × *g* pellet) and in the cytosolic (100,000 × *g* supernatant) fractions prepared from fetal kidneys. Results showed that SLC12A1 was mainly detected in the membrane fractions of the unaffected kidneys, whereas they were weakly detected in the affected kidneys (Fig. 5g). Consistent with this observation, the amount of SLC12A1 in the cytosolic fractions of the affected kidneys was higher than in the unaffected kidneys (Fig. 5g). These results suggested that p.Pro372Leu impairs the apical membrane localization of SLC12A1, which in turn, could affect the transporter activity.

### Identification of a founder individual and surveillance of the risk-allele frequency in the Japanese Black cattle population

**Fig. 6:**
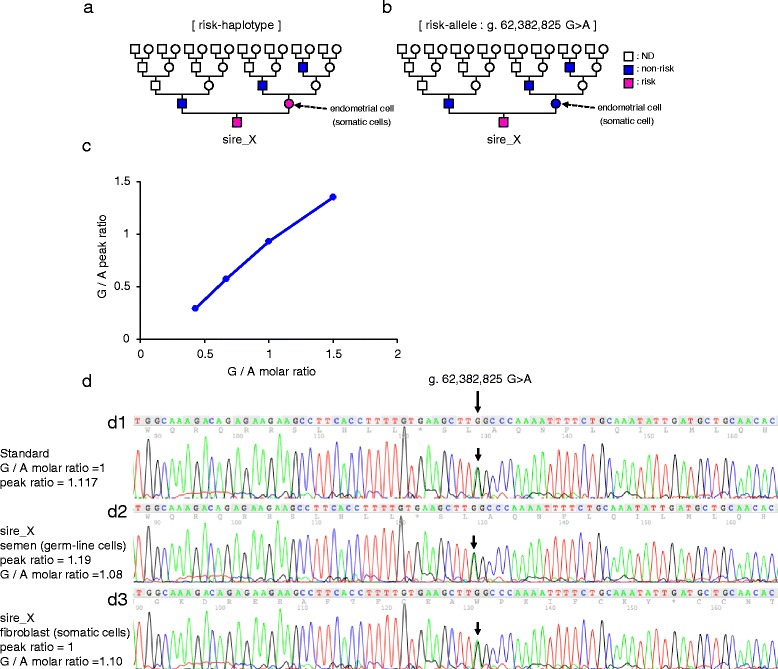
Identification of a founder individual for hydrallantois in Japanese Black cattle. gDNA was extracted from semen (germ-line cells) from sire_X, the father, maternal grandsire, maternal great-grandsire, and from endometrial cells (somatic cells) derived from the uterus of the mother of sire_X. The gDNA was genotyped using GGP Bovine 26 K BeadChip array and g.62382825G > A using PCR. **a**, **b** Non-risk-haplotypes/non-risk-allele carriers, and risk-haplotype/risk-allele carriers are represented with blue-filled symbols and magenta-filled symbols, respectively. Individuals for which we could not determine the haplotype or the allele by genotyping are denoted by empty symbols (ND). **c** Calibration curve of g.62382825G > A. **d** Sequence electropherograms G/A allele molar ratio in gDNA from semen (germ-line cells, **d2**) and gDNA from dermal fibroblast (somatic cells, **d3**) from sire_X was calculated with the ratio of the peak height of G:A alleles in gDNA of the calibration sample (**d1**)

The most effective way to eliminate the causative mutations from a population is to identify a founder individual and then perform targeted genotyping of the related sires used for breeding. Pedigree analysis showed that parents of the nine affected fetuses traced back to sire_X (Fig. 2). To trace back the risk-haplotype and the risk-allele of sire_X, we genotyped the parents, the maternal grandsire, and the maternal great-grandsire of sire_X (Fig. 6a, b) using GGP Bovine 26 K BeadChip array and g.62382825G > A using PCR. gDNA was extracted from the semen (germ-line cells) of sire_X, the father, the maternal grandsire, the maternal great-grandsire, and from the endometrial cells (somatic cells) derived from the uterus of the mother of sire_X (Fig. 6a, b). We found that the mother of sire_X was heterozygous for the risk-haplotype, whereas the father, maternal grandsire, and maternal great-grandsire did not have the risk-haplotype (Fig. 6a), indicating that the risk-haplotype was transmitted from the mother. However, the mother did not have the risk-allele (Fig. 6b), suggesting that a de novo mutation was generated in the germ-line cells of the mother or in the germ-line cells of sire_X with mosaic status.

To determine the composition rate of the risk-allele in germ-line cells and somatic cells of sire_X, we genotyped the risk-allele in semen (germ-line cells) and dermal fibroblast cells (somatic cells) from sire_X and compared the relative abundance of the reference allele to the risk-allele using an allelic imbalance test [34]. Results showed that the risk-allele in sire_X was detected equally as well as the reference allele in both the semen and fibroblasts (Fig. 6c, d), indicating that sire_X was not mosaic for the mutation (g.62382825G > A). Based on all the above findings, a de novo mutation (g.62382825G > A) may have been generated in the germ-line cells, eg, oocyte of the mother, on the risk haplotype, and sire_X was the founder individual of g.62382825G > A (Fig. 6).

### Surveillance of the risk-allele frequency in Japanese Black cattle population

When a recessive disorder is discovered in a population, the risk-allele might already have reached a high frequency in that population. The allele frequency of the risk-allele was 0.004 in the central slaughterhouses, which received animals from locations across Japan, and was 0.052 in the local subpopulation, where hydroallantois mainly occurred and sire_X was reared, respectively (Table 2), indicating that the risk-allele was not widespread in the Japanese Black cattle population throughout Japan. The frequency of the risk-allele in 73 offspring of the carrier sire was 0.285, whereas a homozygote for the risk-allele was not observed (Table 2). Frequency of the genotype significantly deviated from the expected frequency, estimated from the frequency of the risk-allele (chi-square test, *P* = 0.0037), which was consistent with the fetal lethality in hydrallantois.

**Table 2:** Genotypic frequencies of g.62382825G > A in *SLC12A1* in central slaughterhouses, the local subpopulation, and offspring of carrier sires

Central slaughterhouses^a^ (*N* = 1221)					Local subpopulation^b^ (*N* = 1102)					Offspring of carrier sires (*N* = 73)				
	No.	Genotype freq	Exp freq	Exp No.		No.	Genotype freq	Exp freq	Exp No.		No.	Genotype freq	Exp freq	Exp No.
^G/G^	^1212^	^0.993^	^0.993^	^1212.02^	^G/G^	^988^	^0.897^	^0.899^	^1097.96^	^G/G^	^36^	^0.554^	^0.703^	^45.70^
^G/A^	^9^	^0.007^	^0.007^	^8.97^	^G/A^	^114^	^0.103^	^0.098^	^119.78^	^G/A^	^37^	^0.569^	^0.477^	^31.02^
^A/A^	^0^	^0.000^	^0.000^	^0.02^	^A/A^	^0^	^0.000^	^0.003^	^3.27^	^A/A^	^0^	^0.000^	^0.081^	^5.27^
A allele freq = 0.004					A allele freq = 0.052					A allele freq = 0.285				

^a^Central slaughterhouses were in the Tokyo Metropolitan Central Wholesale Market, Tokyo, and Nanko Wholesale Market, Osaka

^b^Individuals were reared in Shimane Prefecture

### Mechanisms of hydrallantois with SLC12A1 (p.Pro372Leu)

**Fig. 7:**
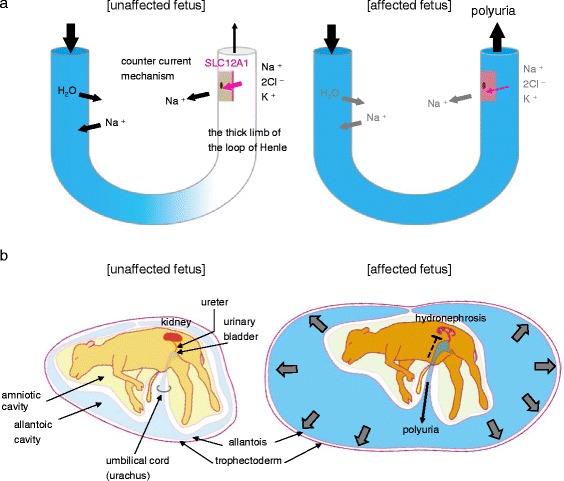
Model of hydrallantois in Japanese Black cattle. **a** SLC12A1 is involved in the countercurrent mechanism in the kidney. In the kidneys of unaffected fetuses (*left panel*, **a**), SLC12A1 is localized at the apical membrane in the thick ascending limb of the loop of Henle and serves as a reabsorption molecule of Na^+^-K^+^-2Cl^−^ in the tubules. Transportation of Na^+^ from the intracellular to interstitial spaces by other transporters leads to an increased osmolality in the interstitial space. The increased interstitial osmolality draws water from the descending thin limb, progressively concentrating the primary urine in the tubules. In the affected fetus kidneys (*right panel*, **a**), SLC12A1 dislocates from the apical membrane to cytosol, in turn, impairing reabsorption of Na^+^-K^+^-2Cl^−^ in the thick limb of the loop of Henle, leading to defective concentration of urine via the countercurrent mechanism. Consequently, the affected fetuses exhibit polyuria. **b** Fetal urine storage in the allantoic cavity of the uterus. At mid-gestation, excessive volumes of allantoic fluid obstruct the outflow of fetal urine into the allantoic cavity (*right panel* **b**, dotted line) due to limited space in the uterus, resulting in hydronephrosis. The maternal abdomen becomes progressively distended and increases the pressure on the internal organs and fetus in the uterus, causing fetal death

In kidneys of the normal fetuses, SLC12A1 reabsorbs Na^+^-K^+^-2Cl^−^ at the apical membrane in the thick ascending limb of the loop of Henle. Transportation of Na^+^ from the intracellular to interstitial spaces by other transporters leads to increased osmolality in the interstitial space, which draws water from the descending thin limb, progressively concentrating the primary urine in the tubules (for review, see [21]) (Fig. 7a). In contrast, the fetuses affected with hydrallantois have impaired Na^+^ reabsorption via dislocation of SLC12A1 from the apical membrane, which wastes Na^+^ in the urine (Fig. 7a). Consequently, the affected fetuses are defective in concentrating urine and exhibit polyuria (Fig. 7a), which accumulates in the allantoic cavity in the uterus (Fig. 7b). Functional mesonephros are completely developed at the mid-late gestation stage as described by Skydsgaard [13] and references therein; thus, the fetal kidneys produce large amounts of urine from around the mid-gestation stage onwards. Therefore, in the affected fetuses, an excessive volume of allantois fluid from around the mid-gestation stage onwards causes an obstruction to the outflow of fetal urine into the allantoic cavity (Fig. 7b, dotted line), resulting in hydronephrosis in the fetal kidneys (Fig. 1f, h). In 1956, Neal also reported three cases of hydrallantois with hydronephrosis in Ayrshire and Guernsey cattle fetuses [11]. Subsequently, the maternal abdomen becomes progressively distended at mid-gestation (Fig. 1b) and the allantoic fluid increases the pressure on the internal organs and the fetus in the uterus, finally causing fetal death.

The dysfunctional *SLC12A1* in humans is responsible for the antenatal form of Bartter syndrome type I [23–26], which is characterized by an accumulation of fetal urine in the amnios through urethra, termed “hydramnios” [23–26]. Based on the structural differences between cattle and human placental tissue [35–37] (for schematic image, see Additional file 3), urine from kidneys in cattle fetuses mainly accumulates in the allantoic cavity through the urachus, termed “hydrallantois” and exhibits slightly different symptoms from the same molecular disorder.

Of particular note, other types of Bartter syndrome (Bartter syndrome types 1–5) and Gitelman syndrome (a subset of Bartter syndrome) are also caused by the mutation of genes encoding proteins that transport ions across the renal cells in the kidneys in humans (Additional file 1: Table S9) [21]; thus, mutations in other renal ion transporters have the potential to cause hydrallantois in cattle. However, other Bartter syndrome-associated genes were not found in the risk-haplotype region and we did not find any mutations in other Bartter syndrome-associated genes (Additional file 1: Table S9) that were compatible with the recessive inheritance. These findings further support the hypothesis that *SLC12A1* (﻿g.62382825G > A,﻿ p.Pro372Leu) is the causative gene for hydrallantois in Japanese Black cattle.

## Conclusion

In this study, we mapped the shared haplotype of the hydrallantois-affected fetuses using autozygosity mapping and identified a de novo mutation in *SLC12A1* gene in an elite sire that was associated with hydrallantois in Japanese Black cattle. In the kidneys of hydrallantois-affected fetuses, the *SLC12A1* mutation was observed to impair the reabsorption of Na^+^-K^+^-2Cl^−^ in the thick limb of the loop of Henle via dislocation of SLC12A1 from the apical membrane, leading to defective concentration of urine via the countercurrent mechanism. Consequently, the affected fetuses exhibited polyuria and the pregnant cows showed hydrallantois. Surveillance of the risk-allele indicated that the carriers were not widespread throughout the Japanese Black cattle population and identified a founder individual; thus, we are able to effectively manage the disorder in the population.

## Methods

### Collection of animal samples with phenotypic data and biological materials

Between October 2014 and April 2015, we obtained biological material and clinical information with pedigree records of nine Japanese Black cattle diagnosed with hydrallantois (for diagnosis criteria, see [1]). Abdominal circumference was measured from between the 13th thoracic vertebra and the 4th lumber vertebra to the umbilicus. Between 5.1 months (158 days) to 9.4 months (280 days) of gestation, parturition in eight hydrallantois cases was induced by prostaglandin F2α following the standard national guidelines, and one case was that of stillbirth. Gross necropsy of the affected fetuses was performed by veterinarians in the Shimane Regional Livestock Hygiene Service Center. Samples of the unaffected fetuses were obtained from the slaughterhouse (Shimane Prefecture). For DNA analysis, tissue samples (ear skin: 2 cm × 2 cm) of the affected fetuses were collected using surgical scissors and stored in 70 % ethanol until DNA extraction. For histopathological examination, the tissue samples were fixed with 10 % neutral-buffered formalin and embedded in paraffin using standard procedures. Sections (3 μm) were stained with hematoxylin and eosin.

### Determining the sex of affected fetuses

Y chromosome specific primers are shown in Additional file 1: Table S1. PCR was performed as described by McDaneld et al. [38].

### Extraction of gDNA from ear skin, semen, and blood

Tissue samples were stored in 70 % ethanol until DNA extraction. After washing with Dulbecco’s phosphate-buffered saline (PBS) (Sigma, Cat. #D8567), gDNA was extracted using a lysis buffer (500 μg/mL Proteinase K [Merck, Cat. #1.24568.0500], 40 mM dithiothreitol [Wako, Cat. #048-29224], 0.08 % SDS and 1 × PCR buffer [Takara, Cat. #R001A]) at 50 °C for 16 h and then extracted with phenol-chloroform, and precipitated with ethanol using the standard procedures [39]. Semen samples were washed with 0.11 M sodium citrate and PBS twice before gDNA extraction. gDNA of whole blood was extracted using the Easy-DNA Purification Kit (Invitrogen, Cat. #K1800-01). DNA quality was confirmed by agarose electrophoresis.

### Autozygosity mapping using Bovine 26 K BeadChip

A total of 23 DNA samples (six from the affected fetuses and 17 from the unaffected calves; the unaffected calves being the offspring sired by fathers of the affected fetuses) were genotyped using the GGP_SuperLDv2-0 26 K BeadChip (GeneSeek, Cat. #15052088), which included probes for 26,151 SNPs. The UMD3.1 assembly [40] was used to map the position of the SNPs. After eliminating the call rates > 99 %, autozygosity mapping was performed using the ASSIST program [16].

### Identification of CNVs using BovineHD BeadChip Array

To determine whether the large structural variants, such as copy number variations (CNVs) in the risk-haplotype region were involved in hydrallantois, we genotyped six affected fetuses, the parents of the nine affected fetuses, and sire_X using the Illumina BovineHD BeadChip Array (Illumina, Cat. #WG-450-1002), which contained 735,293 autosomal SNPs with an average marker interval of 3.416 kb [15], according to the manufacturer instructions. CNVs were detected using PennCNV software (version June 2011) [41, 42], which incorporates factors including Log R ratio, B allele frequency, marker distance, and the population frequency of B allele into a hidden Markov model. We used the CNVs database of 1481 Japanese Black cattle that were genotyped using the Illumina BovineHD BeadChip Array [17].

### Haplotype phasing of SNPs

Genotypes of SNPs were phased using BEAGLE3.3.2 software [43, 44].

### Exome sequencing

Two micrograms of gDNA in 50 % glycerol/1 × Low TE buffer (Life Technologies, Cat. #12090-015) from each individual was sheared to 250–450 bp by using Nebulizers (Life Technologies, Cat. #K7025-05). Individual paired-end libraries were prepared using KAPA library preparation kit (KAPA, Cat. #07 137 923 001), captured using a custom SeqCap EZ developer library for whole exomes [45], and sequenced on an Illumina HiSeq2500 (2 × 101 bp). Initial sequence processing, base-calling, and de-multiplexed sequence reads were performed using Illumina CASAVA v.1.8.0. Sequence reads (FASTQ) for each sample were aligned to the UMD3.1 assembly of the bovine genome using Burrows-Wheeler Aligner [46]. Duplicates were marked using Picard [47], and indexed, merged, and sorted using SAMtools [48]. The GATK utility UnifiedGenotyper was used to identify and extract the SNPs and Indels [49]. Functional variant annotation and effect prediction was performed using snpEff [50], PolyPhen-2 [18], and Variant Effect Predictor (VEP) [51] based on gene annotation of the UMD3.1 assembly of the bovine genome.

### Localization of SLC12A1 in kidneys of cattle fetuses

Kidney samples were collected in cryotubes and snap frozen in liquid nitrogen. Tissues were trimmed using cryostat blade (Leica, Cat. #14035838926) and then embedded in the OCT compound and frozen. Sections (15 μm) were fixed in 4 % paraformaldehyde at 4 °C for 15 min. After blocking with 10 % goat serum/PBS for 1 h, SLC12A1 and Tamm-Horsfall glycoproteins were detected with rabbit anti-SLC12A1 (2.5 μg/mL, Abcam, Cat. #ab191315) and sheep anti-Tamm-Horsfall glycoprotein (1:1000, Millipore, Cat. #AB733) at 37 °C for 1 h and subsequently at 4 °C for 16 h. Immunoreactivity was detected with TRITC-anti-rabbit IgG antibody (Jackson ImmunoResearch, Cat. #025-144-111) and FITC-anti-sheep IgG antibody (Jackson ImmunoResearch, Cat. #213-542-177). Both the 1st and 2nd antibodies were diluted with solution A of Can Get Signal immunostain (Toyobo, Cat. # NKB-501). Sections were counter-stained with a DAPI (Molecular Probe, Cat. #D1306) and examined under a confocal microscope (FV1000; Olympus Optical). Confocal images were analyzed by plot profile (Image J) [52] to semi-quantify the relative fluorescence intensity.

### Expression analysis

For real-time quantitative PCR, we extracted total RNA from kidneys of 25-month-old cattle and kidneys from two unaffected and two affected fetuses 43–51 cm CRL (approximately 160 days of gestation [29]) using RNeasy Mini Kits (QIAGEN, Cat. #74104). Total RNA was treated with DNase I. cDNA was synthesized from 50 ng total RNA using the ReverTra Ace-α Kit (TOYOBO, Cat. #FSK-101) with random primers, according to the manufacturer instructions. The *SLC12A1* gene was detected with the following primers and probe: forward, 5′-gacctgctctcttggatataactca-3′; reverse, 5′-tgccatgccactgttcatctc-3′; and probe, 5′-acacagcttgcgtggtcccacaaagac-3′. Real-time PCR was performed on a 7900HT Real-Time PCR System (Applied Biosystems) using the comparative Ct method with glyceraldehyde-3-phosphate dehydrogenase (*GAPDH*) mRNA serving as the internal control.

### Preparation of membrane and cytosolic fractions

Fetal kidney tissues were homogenized in a 0.25 M sucrose solution containing 5 mM Tris-HCl, pH 7.5 and protease inhibitor (Roche, Cat. #14512300) using a Potter-Elvehjem homogenizer. Nuclei and unbroken cells were removed by centrifugation (600 *g* for 10 min). Membrane and cytosolic fractions were centrifuged at 100,000 × *g* for 1 h at 4 °C in an Optima ultracentrifuge (Beckman, Cat. #TL-100). Expression of SLC12A1 was detected with western blotting using an anti-SLC12A1 antibody (Abcam, Cat. #ab171747, 1:1000). Immunoreactivity was detected with a horseradish peroxidase-conjugated donkey anti-rabbit IgG antibody (Jackson ImmunoResearch, Cat. #711-036-152, 1: 10,000) and the ECL Prime Western Blotting Detection Reagent (GE Healthcare, Cat. #RPN2232). Chemiluminescence was detected with an ImageQuant LAS 4000 (GE Healthcare).

### Allelic imbalance test

To quantify the allelic imbalance of *SLC12A1*, we designed PCR primers for g.62382825G > A (the details of primer are provided in Additional file 1: Table S5). We used 10 ng of template gDNA from dermal fibroblasts or semen from sire_X for PCR amplification with TaKaRa Ex Taq HS DNA polymerase (TaKaRa, Cat. #RR006). The PCR product was directly sequenced and purified with the CleanSEQ system (Agencourt, Cat. #A29154). Peak heights at polymorphic sites were quantified using PeakPicker 2 software [34]. To estimate the molar ratio of the SNP at peak height, calibration curves were generated from data obtained by mixing varying amounts of gDNA from individuals with the homozygous risk-allele and the homozygous reference allele.

### Surveillance of risk-allele frequency in Japanese Black cattle

To survey the risk-allele frequency in Japanese Black cattle in Japan, we genotyped 1221 animals from two central slaughterhouses, which received animals from locations across Japan (Tokyo Metropolitan Central Wholesale Market, Tokyo, and Nanko Wholesale Market, Osaka) between 2003 and 2008, and 1102 animals that were reared in the local subpopulation (Shimane Prefecture) between 1999 and 2015, where hydroallantois mainly occurred and sire_X was reared. The g.62382825G > A SNP was genotyped by directly sequencing the PCR products using PCR primers for g.62382825G > A (the detail of the primer used are shown in Additional file 1: Table S5). The PCR products were sequenced using the forward primer and the BigDye Terminator v.3.1 Cycle Sequencing Kit (Applied Biosystems), followed by electrophoresis using an ABI 3730 sequencer (Applied Biosystems) and genotyping using SeqScape software, V2.5 (Applied Biosystems).

## Additional files

Additional file 1: Table S1. Primer information for Y chromosome and *GAPDH*. **Table S2.**The shared haplotype of hydrallantois in the affected fetuses. Positions are based on the UMD3.1 assembly of the bovine genome. **Table S3.**Detailed features of CNVs in the critical region of hydrallantois in 1481 Japanese Black cattle. Positions are based on the UMD3.1 assembly of the bovine genome. CNVs were detected from 1481 Japanese Black cattle [17] using the Illumina BovineHD BeadChip Array [15]. ^1^ loss type of CNV is the deleted type; gain type of CNV is the duplicated type. ^2^ Haplotypes of 1481 animals were determined by Beagle software. **Table S4.**Genes within the critical region of hydrallantois in Japanese Black cattle. Positions are based on the UMD3.1 assembly of the bovine genome. **Table S5.**Primer information for g.62111230G > A and g.62382825G > A. Positions are based on the UMD3.1 assembly of the bovine genome. **Table S6.**Transcript and protein ID of bovine *SLC12A1*. **Table S7.**Genotypes of two variants validated using Sanger sequencing. ^1^ Phenotype was diagnosed by veterinarians following the standard guidelines and textbooks (for diagnosis criteria, see [1]). ^2^ Haplotypes were genotyped by GGP_SuperLDv2-0 26 K BeadChip and determined by Beagle software. ^3^ N = Non-risk-haplotype; Risk = risk-haplotype. **Table S8.**Primer information for exon of *SLC12A1*. Positions are based on the UMD3.1 assembly of the bovine genome. **Table S9.**Bartter syndrome-associated genes and their positions on the UMD3.1 assembly of the bovine genome. Positions are based on the UMD3.1 assembly of the bovine genome. ^1^ Bartter syndrome type 4B is caused by simultaneous mutations in both the *CLCNKA* and *CLCNKB* genes. ^2^ *CLCNKB* is not identified or mapped on the UMD3.1 assembly of the bovine genome. ^3^ Gitelman syndrome is formerly considered a subset of the Bartter syndrome. (XLSX 50 kb) Additional file 2: Sex of affected fetuses. PCR products with 119 bp and 216 bp were amplified using the primer pairs for the Y chromosome and *GAPDH*, respectively. Lanes 1 to 9: affected fetuses; lanes 10 and 11: cows; and lanes 12 and 13: sires. M: 100 bp ladder marker. (PPTX 902 kb) Additional file 3: Schematic images of placental tissues in cattle and humans. (A) Cattle fetus and the placental tissue. (B) Human fetus and placental tissue. The images have been adapted from Leiser and Kaufmann [35] and Fernandes et al. [36]. Magenta dotted arrows represent dominant flow path of urine in the affected fetuses in “hydroallantois” cattle and “hydramnios” humans, respectively. (PPTX 2449 kb)

## Abbreviations

BTA: (*Bos taurus*) chromosome
CNV: Copy number variation
CRL: Crown-rump length
PCR: Polymerase chain reaction
SNP: Single nucleotide polymorphism
