# Supplementary material for: A missense mutation in solute carrier family 12, member 1 (SLC12A1) causes hydrallantois in Japanese Black cattle
Source: BMC Genomics. 2016 Sep 9;17(1):724. doi: 10.1186/s12864-016-3035-1 (PMC5016959; doi:10.1186/s12864-016-3035-1)
Supplement: Additional file 2: — Sex of affected fetuses. PCR products with 119 bp and 216 bp were amplified using the primer pairs for the Y chromosome and GAPDH, respectively. Lanes 1 to 9: affected fetuses; lanes 10 and 11: cows; and lanes 12 and 13: sires. M: 100 bp ladder marker. (PPTX 902 kb) [file 12864_2016_3035_MOESM2_ESM.pptx]

## Slide 1
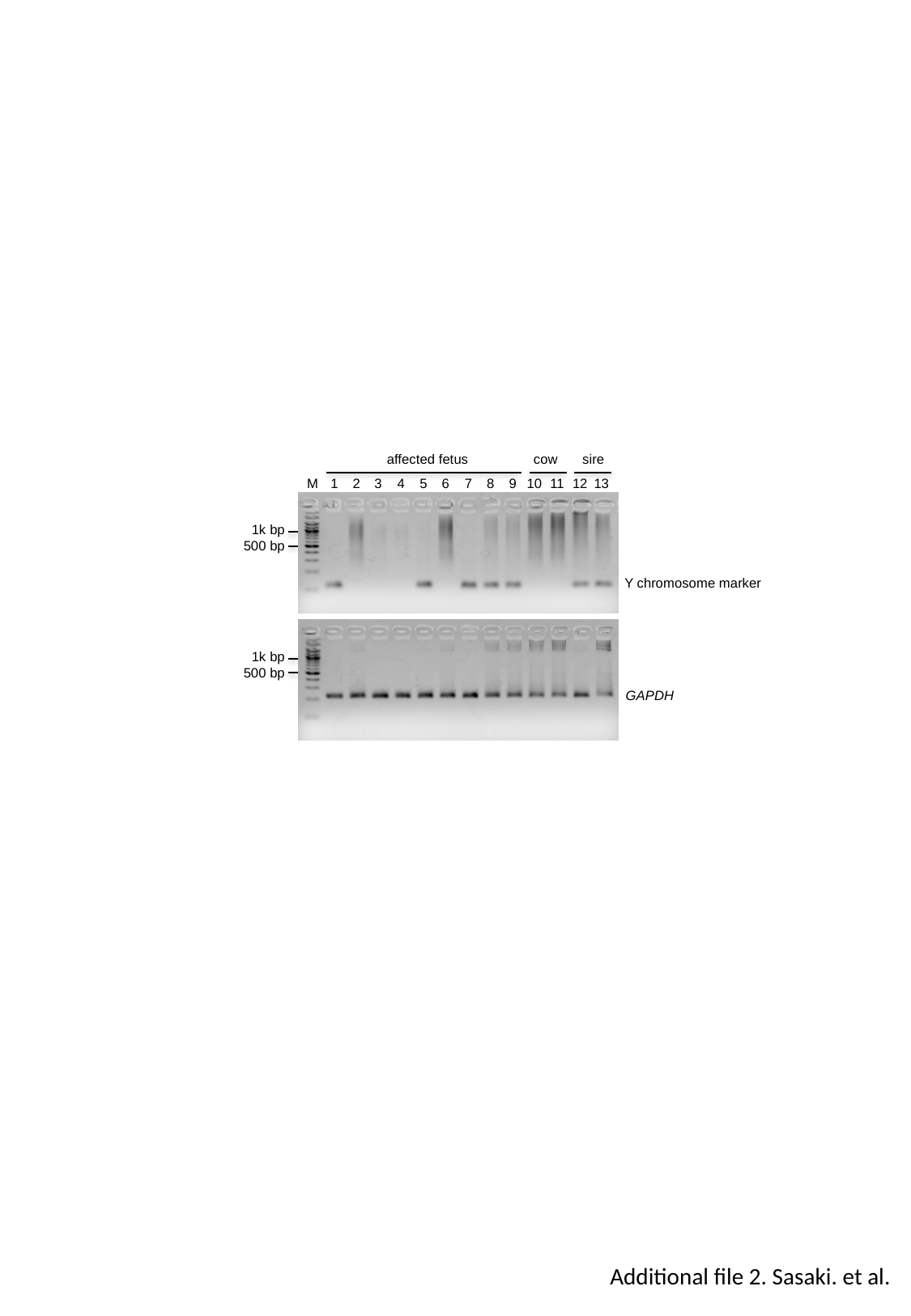

affected fetus
cow
sire
M
1
2
3
4
5
6
7
8
9
10
11
12
13
1k bp
500 bp
Y chromosome marker
1k bp
500 bp
GAPDH
Additional file 2. Sasaki. et al.
