# Supplementary material for: A missense mutation in solute carrier family 12, member 1 (SLC12A1) causes hydrallantois in Japanese Black cattle
Source: BMC Genomics. 2016 Sep 9;17(1):724. doi: 10.1186/s12864-016-3035-1 (PMC5016959; doi:10.1186/s12864-016-3035-1)
Supplement: Additional file 3: — Schematic images of placental tissues in cattle and humans. (A) Cattle fetus and the placental tissue. (B) Human fetus and placental tissue. The images have been adapted from Leiser and Kaufmann [35] and Fernandes et al. [36]. Magenta dotted arrows represent dominant flow path of urine in the affected fetuses in “hydroallantois” cattle and “hydramnios” humans, respectively. (PPTX 2449 kb) [file 12864_2016_3035_MOESM3_ESM.pptx]

## Slide 1
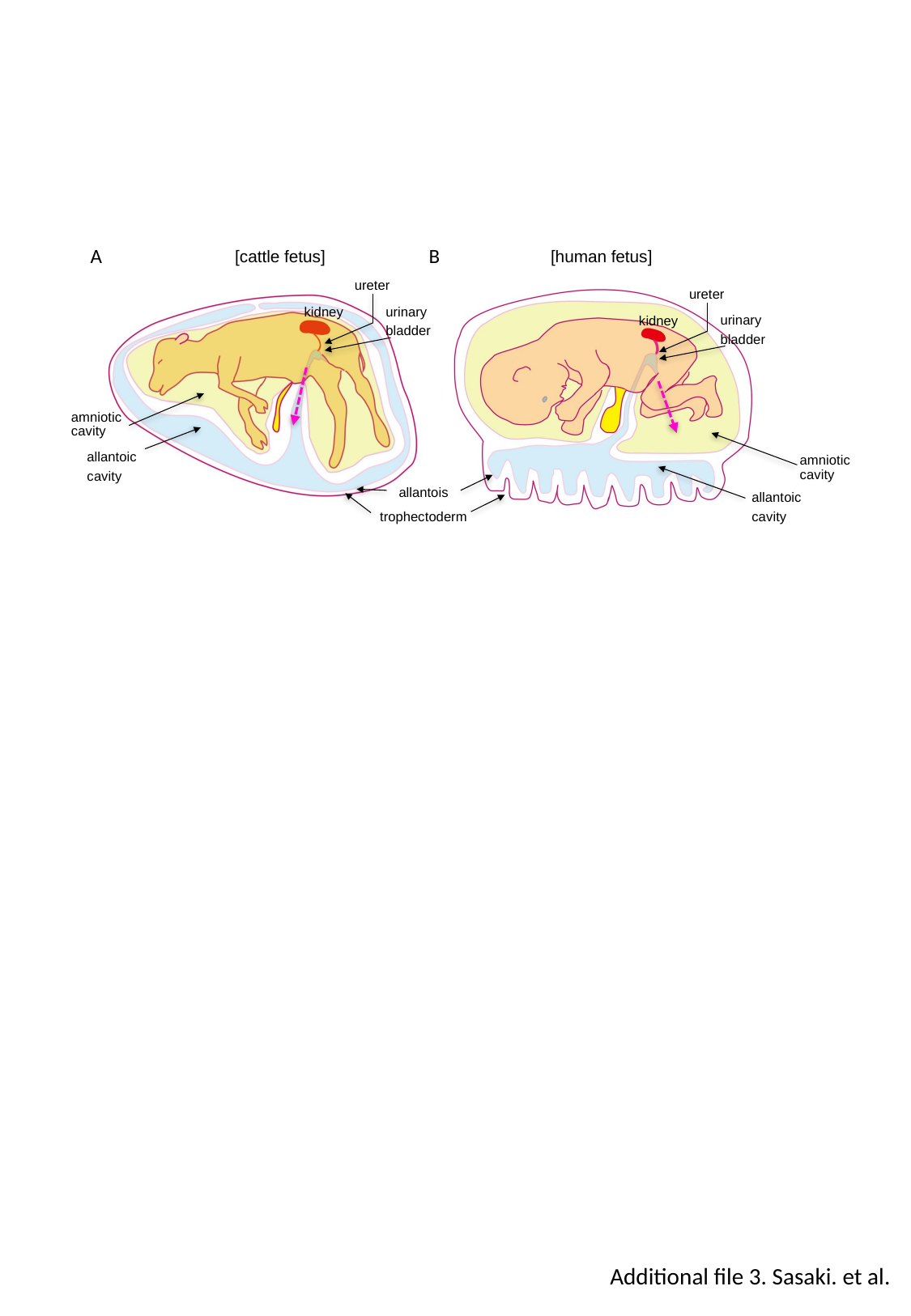

[cattle fetus]
[human fetus]
A
B
ureter
ureter
urinary
bladder
kidney
urinary
bladder
kidney
amniotic
cavity
allantoic
cavity
amniotic
cavity
allantois
allantoic
cavity
trophectoderm
Additional file 3. Sasaki. et al.
